# Abstracts and Selections

**Published:** 1909-06

**Authors:** 


### Abstracts and Selections.

### Evolution.

BY LANGDON SMITH.

When you were a tadpole and I was a fish,
  In the paleozoic time,
And side by side on the sluggish tide,
  We sprawled through the ooze and slime,
Or skitted with many a caudal flip
  Through the depths of the Cambrian fen,
My heart was rife with the joy of life,
  For I loved you even then.

Mindless we lived, mindless we loved,
  And mindless at last we died,
And deep in the rift of the Caradoc drift,
  We slumbered side by side.
The world turned on in the lathe of time,
  The hot sands heaved amain,
Till we caught our breath in the womb of death,
  And crept into life again.

Then we were amphibians, scaled and tailed,
  And deaf as a dead man's hand,
We sprawled at ease beneath the trees,
  Or scrawled through the mud and sand,
Croaking and blind, with our three-clawed feet,
  Writing a language dumb.
With never a spark in the empty dark,
  To hint of a life to come.

Yet happy we lived, happy we loved,
  And happy we died once more,
And our forms were rolled in the clinging mould
  Of a Neocomian shore,
And the Aeons came, and the Aeons fled,
  Till the sleep that bound us fast
Was driven away by the dawn of day,
  And the night of death was past.

Then light and swift through the jungle trees
  We swung in our airy flights,
Or dreamed in the balm of the fronded palm
  In the hush of those moonlight nights,
And, oh, what beautiful years were these
  Where our hearts clung each to each,
And life was frilled, and the senses thrilled
  With the first faint dawn of speech.

Thus, life by life, and death by death,
  We passed through the cycles strange,
And love by love, and breath by breath,
  We followed the chain of change.
Till there came a time in the law of life,
  And o'er the reeking sod,
The shadows broke and the soul awoke
  In the first dim dream of God.

Then I was thewed like an Auric bull,
  And tusked like the great cave bear,
And you, my sweet, from head to feet,
  Were gowned in your glorious hair.
Deep in the gloom of the fireless cave,
  When the night fell over the main,
And the moon hung red o'er the river bed,
  We numbered the bones of the slain.

For we lived by blood and the right of might,
  Ere the human laws were drawn,
For the age of sin did not begin,
  Till our brutish tusks were gone,
And that was a million years ago,
  In a time when no man knows,
Yet here tonight in the mellow light
  We sit at Delmonico's.

Your eyes are as deep as the Devon Springs,
  Your hair is as black as jet,
Your years are few, your life is new,
  Your soul untried, and yet,
Our trail is on the Kimmeridge clay,

In the scarp of the Birbeck flags,
We had left our bones in the Bagshot stones
And deep in the Coraline crags.

Our love is old, our life is old,
And death shall come again,
Should he come today, what man may say,
That we shall not meet again?
Then as we sit at our dinner here,
O'er many a dainty dish,
Let us drink anew to the time when you
Were a tadpole, and I a fish.

*—Pacific Medical.*

---

LYDSTON'S PARODY.

When you were a lobster, and I was a peach,
In our youth's roseate time,
And side by side on Broadway's tide we
Blew the froth from the festive stein,

Or staggered with many a stag, through
The haunts of dissolute men,
I wasn't next to the ways of life, and you
Were a sucker, even then.

Heedless we lived and heedless we loved,
And heedless at last we died,
And deep in a hole in the Potter's Field
They buried us, side by side.

The town rolled on, with her hot old time,
And Broadway heaved amain,
But we caught no breath from the womb of death,
For we never peeped again.

So we can't take lunch at Delmonico's,
'Tis a joint that's quite out of reach,
And we'll not drink anew to the time when you
Were a lobster, and I was a peach.

*—Pacific Medical.*

Fourth of July and Tetanus.—Once upon a time there was a man who pretended to possess a classical education. This is how he uttered a well-known Latin phrase: "How *tempus* does *fugit!*" Before we are aware of it, the "glorious Fourth" will be with us, with its list of casualties, preventable in nearly every instance. Prevention, prevention, and again prevention, should be the watchword. A "sane" fourth of July really means a day without pyrotechnics or firearms of any kind. As this is too much to expect, our next attention should be directed towards the proper treatment of all punctured wounds. Doland, in *Northwest Medicine* (May), speaks of the subject with the air of one having authority:

"Prevention is the keynote of this disease (tetanus). As Welch has pointed out, the antitoxin of tetanus has proved to be rather a disappointment, for the amount needed to save the patient from the toxin being manufactured in his body by the bacilli increases so rapidly and enormously with the day or hour of the disease, as to make the dosage, which increases with millions of times where that of diphtheria antitoxin increases but tenfold, a matter of difficulty and uncertainty. Nocard also calls attention to the fact that the existence of tetanus is unknown until there is sufficient toxemia to produce spasms, and that, therefore, it is impossible to attack the disease in its inception; we are obliged to meet on the same grounds as diphtheria in the later days of the disease, a time when it is well known that the chances of its improvement are greatly lessened.

"Hence, in all punctured wounds we should open them freely and cleanse then thoroughly, preferably curetting and then lightly packing them, so that the atmospheric air has access to the depths of the wound. Do not use carbolic acid, or any substance that will leave a coagulum and thus shut out the air. In patients with fourth of July wounds, we should, in addition, inject an immunizing dose of antitoxin at the site of the injury. These measures would best be carried out under a general anesthetic."—*Lancet-Clinic.*

---

The Medical Era's Gastro-Intestinal Editions.—During July and August, the *Medical Era* of St. Louis, Mo., will issue its annual series of issues devoted to gastro-intestinal diseases. The July number will take up the usual bowel disorders of hot weather and the August will be devoted entirely to typhoid fever. These issues always attract considerable attention. The editor will forward copies to physicians applying for same.

PATIENTS who show a progressive loss of vocal power should be examined most carefuly for an intralaryngeal condition. An acute aphonia may be due to an inflammatory condition or paresis of one cord; alcoholism, syphilis, tuberculosis and malignant disease bring on a chronic condition. Two most important causes of chronic laryngitis are thickening due to an old inflammatory process and the presence of a small, hard, nodular tumor on one of the cords, *e. g.*, fibroma.—*Journal of American Surgery.*

---

A SCIENTIFIC PRIEST ON THE DANGERS OF HOLY WATER.—The sanitary dangers lurking in "holy water" have often been referred to by medical men. They have recently been scientifically studied by a monk, Fr. Augustin Gemelli, who is himself a highly qualified medical man. He publishes his results in the *Scuola Cattolica.* Each cubic centimeter of the holy water in the basins in the church of Santa Croce, Turin, taken from the surface contained 150,000 microbes, while a cubic centimeter taken from the bottom contained no less than 6,000,000 microbes. He injected this water into animals and found that it always killed them, the causes of death being tuberculosis, colitis, or diphtheria. He does not think a daily cleansing with corrosive sublimate sufficient, but recommends a new form of holy water receptacle so constructed that persons instead of dipping their fingers into it can obtain three drops of water by pressing a button. A vessel of this nature has been placed in the church of Vergiate, Milan. Fr. Gemelli turned his attention also to the grilles in the confessional boxes. Water which had been used for washing these only contained 25 microbes per cubic centimeter and when injected into animals only proved fatal to 10 per cent of them.—*London Lancet.*

